# Commentary: The causal effect of hypertension, intraocular pressure, and diabetic retinopathy: a Mendelian randomization study

**DOI:** 10.3389/fendo.2024.1399640

**Published:** 2024-08-29

**Authors:** Youqian Zhang, Yulan Chen, Fang Peng

**Affiliations:** Health Science Center, Yangtze University, Jingzhou, Hubei, China

**Keywords:** Mendelian randomization, hypertension, diabetic retinopathy, intraocular pressure, GWAS (genome-wide association study)

Diabetic retinopathy (DR) is a principal microvascular complication of diabetes mellitus (DM) and remains one of the leading global causes of preventable blindness and vision impairment (1, 2). Numerous studies have focused on the association between hypertension and intra-ocular pressure (IOP) with DR. However, due to the limitations of traditional research methodologies, the causal relationship between these factors has remained unclear. Consequently, we were particularly interested in the paper published by Wang et al. (3), which, for the first time, employs Mendelian randomization (MR) to confirm the causal links between hypertension, IOP, and an increased risk of DR.

To ensure the robustness of MR analysis results, research design and methodology must be rigorous. Although Wang et al.’s study holds some merit, issues such as dataset selection, a high rate of sample overlap, and violation of the two-sample MR principle weaken the study’s outcomes, leading to potential false positives. First, using the same individuals to estimate the association between genetic variables, exposures, and outcomes can bias causal effect estimates, especially inflating the statistical significance—a phenomenon known as “sample overlap bias” (4).Two-sample MR employs data from two independent samples to effectively circumvent this issue. However, it is noteworthy that the hypertension (55,917ncase and 162,837 ncontrol) and DR (14,584ncase and 202,082 ncontrol) phenotype data selected by the authors from the FinnGen consortium have an astonishingly high overlap rate of 99%. Additionally, the manuscript contains inaccuracies in the description of the phenotype sample sizes used.

A second major issue concerns the selection of phenotypes. The choice for the IOP phenotype appears unreasonable and deliberate, with the authors selecting data for the left eye’s IOP (ukb-b-12440) while overlooking the right eye’s IOP data (ukb-b-14146), which is not mentioned in the original text. This not only confuses the concept of IOP but may also be a deliberate choice due to positive results. Importantly, the data selected for MR studies should be the most recent and comprehensive. However, the authors selected the R5 version of the FinnGen dataset released in 2021 for the data on DR, rather than utilizing the latest R9 version. For the IOP data, the most comprehensive and largest GWAS study to date by Anthony P Khawaja’s team should be selected (139,555 individuals) (5). This dataset integrates data from the UK biobank (UKB), EPIC-Norfolk, and the International Glaucoma Genetics Consortium (IGGC), rather than relying solely on data from the UKB as used by the authors.

The study also suffers from a series of minor issues. The authors’ description of the sensitivity analyses is ambiguous, particularly in the detection of horizontal pleiotropy. They vaguely reported *P*-values without specifying whether these pertained to MR-Egger regression or the MR-PRESSO Global test. Additionally, it is unclear whether outliers were excluded from the analysis. Furthermore, while calculating the F-statistic to evaluate weak instrumental variables is commendable, considering that most genetic variations explain only a small part of phenotype variability, statistical power becomes a significant challenge in MR analysis (6). The authors failed to consider power calculations. Regarding the third assumption of MR analysis, which is primarily ensured through the MR-Steiger test, the authors did not adequately address this aspect. Given these substantial flaws in various aspects of the study, a re-analysis and re-evaluation of the results are recommended to ensure the research’s robustness and validity.

**Figure 1:**
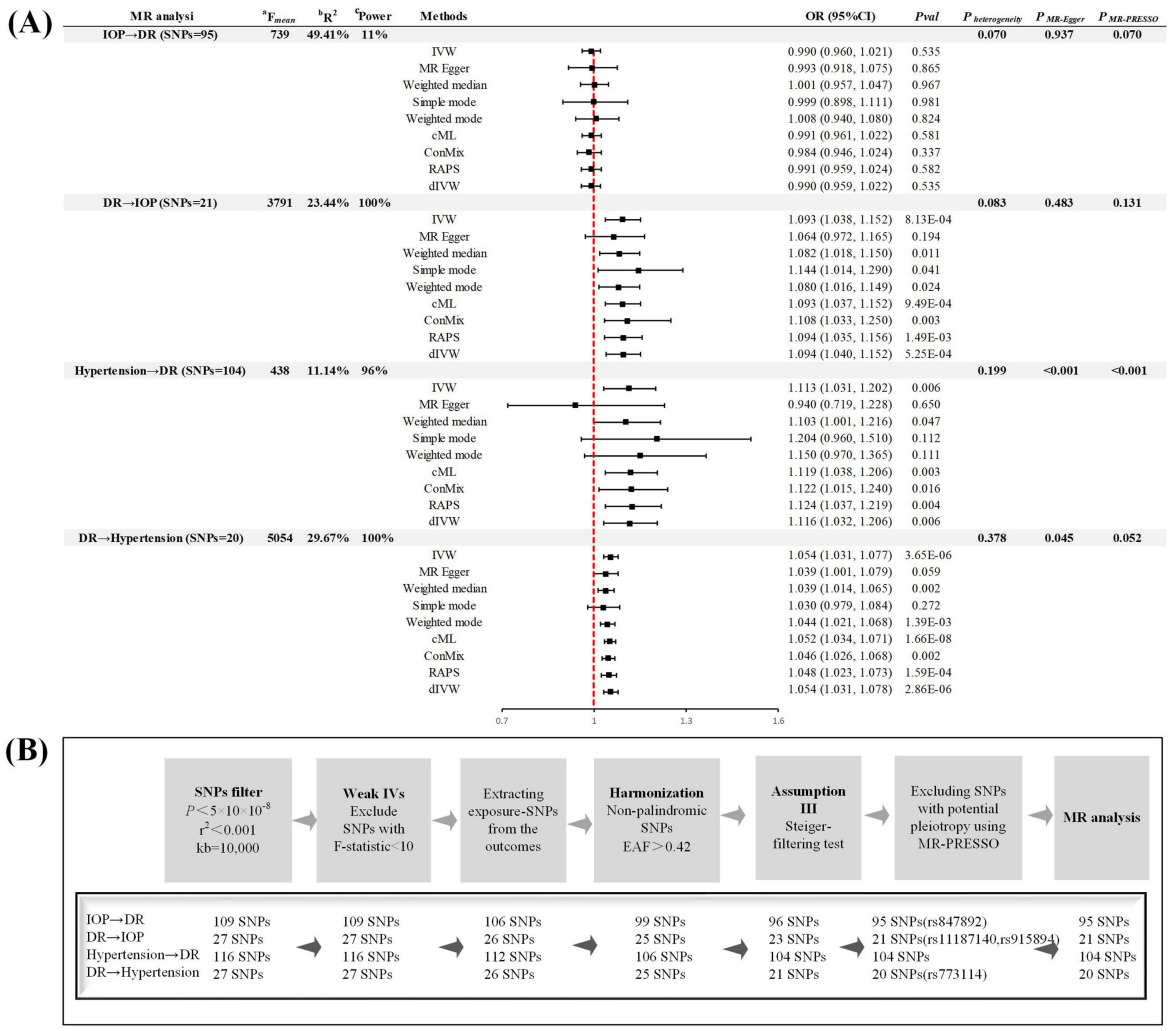
Study design **(A)** Summary of forward and reverse Mendelian randomization analysis. **(B)** Instrumental variable screening process. MR, Mendelian randomization; DR, diabetic retinopathy; IOP, intra-ocular pressure; SNP, single nucleotide polymorphism; IVW, inverse-variance weighted; RAPS, robust adjusted profile score; cML, constrained maximum likelihood; dIVW, debiased inverse-variance weighted; ConMix, contamination mixture; MR-PRESSO, MR Pleiotropy Residual Sum and Outlier; OR, odd ratio; CI, confidence interval; IV, instrumental variable; EAF, effect allele frequency. ^a^F = ((*n*−*k*−1)/*k*)(*R^2^*/(1− *R^2^*)), where R^2^ represents the proportion of exposure variance explained by the SNPs, N denotes the GWAS sample size, and k = 1 reflecting the individual SNP analysis. ^b^R² (the proportion of explained variance) was calculated employing the formula 2×MAF×(1-MAF)×beta², wherein MAF represents the minor allele frequency of each specified SNP. ^c^Power, greater than 80% is considered to be used for strong statistical power, as calculated by an online tool (https://sb452.shinyapps.io/power/).

By acquiring the latest genome-wide association studies (GWAS) data from the FinnGen consortium R9 version (7), UKB (8), and the team led by Anthony P. Khawaja (5), we mitigated biases due to sample overlap (**Table 1**). Building on the methodology proposed by Zhou et al., we have expanded our analysis to ensure the robustness of our findings. This included employing MR-PRESSO to remove outliers and control for horizontal pleiotropy, as well as utilizing methods such as debiased inverse-variance weighted (dIVW), robust adjusted profile score (RAPS), constrained maximum likelihood (cML), and contamination mixture (ConMix) to manage potential pleiotropy and biases. The accuracy of our analysis was further enhanced by calculating R^2^, power, and incorporating the MR-Steiger test. Specifically, each instrumental variable (IV) passed the MR-Steiger test, demonstrating directional consistency as “TRUE”. The MR-PRESSO analysis identified and excluded 4 outliers, significantly minimizing the bias due to horizontal pleiotropy. In the primary method, after Bonferroni correction (*P* < 0.05/4), we identified three significant causal associations, all of which possessed sufficient statistical power to detect the relationships (Power > 95%) (**Figure 1**). In the sensitivity analyses (**Figure 1**), the MR-Egger test did not detect any evidence of pleiotropy (*P* > 0.05). Apart from the analysis of hypertension on DR, both Cochrane’s Q and MR-PRESSO analyses failed to find any evidence of horizontal pleiotropy or heterogeneity (*P* > 0.05), affirming the robustness of our findings. Despite the presence of pleiotropy and heterogeneity evidence in the analysis concerning hypertension’s effect on DR, the application of a random-effects model and supplementary methods such as dIVW, RAPS, CML, and ConMix provided consistent evidence. This approach maximally reduced bias due to pleiotropy, demonstrating the robustness of our results. The leave-one-out analysis further confirmed that the overall causal effect was not driven by any single SNP. The observed discrepancies primarily stem from the original study’s failure to rigorously select phenotypes for analysis and control the study design in the initial MR analysis.

**Table 1:** Detailed information of data sources.

Phenotype	Definition/diagnostics	Adjustments	Ref	Consortium	Ancestry	Participants
IOP	Ocular response analyzer (ORA, Reichert Inc.) and goldmann applanation tonometry. For individuals reporting usage of IOP-lowering medication, measured IOP was divided by 0.7 based on average IOP reduction achieved with medication.	Age, sex, 5 genetic principal components, and genotyping batch	29785010	Anthony P Khawaja' study	EUR	139,555 individuals
DR	ICD-10-H36.00, H36.02, H36.03, and ICD-9-362.0	Age, sex, and up to 20 genetic principal components	36653562	FinnGen (R9)	EUR	10,413 cases and 308,633 controls
Hypertension	Self-reported, ICD-10-I10	Age, sex, 10 genetic principal components, and genotyping batch	36402876	UKB	EUR	77,977 cases and 330,366 controls

UKB, UK Biobank; IOP, intra-ocular pressure; DR, diabetic retinopathy; EUR, European; ICD, international classification of diseases.

In conclusion, our reanalysis corroborates Wang et al.’s findings of a bidirectional causal relationship between DR and hypertension. Contrary to the original findings, our analysis suggests that IOP is not a risk factor for DR; instead, a causal relationship exists where DR leads to elevated levels of IOP. Although the original study made significant contributions to exploring the complex relationship between IOP, hypertension, and DR, our application of the latest GWAS data, expanded analytical methods, and strict adherence to MR principles have considerably enhanced the robustness of the research. Selecting precise datasets and following rigorous analytical principles are crucial for uncovering potential causal links between diseases in future studies.
